# Supplementary material for: Epidemiology of Dementia in China in 2010–2020: A Systematic Review and Meta-Analysis
Source: Healthcare (Basel). 2024 Jan 28;12(3):334. doi: 10.3390/healthcare12030334 (PMC10855047; doi:10.3390/healthcare12030334)
Supplement: Supplementary file 1 [file healthcare-12-00334-s001.zip › healthcare-2795189-supplementary.pdf]

File SI. Search strategy in databases

Search terms	
1. 阿尔茨海默 (Alzheimer) AND	发病率 incidence
	发生率 incidence
	患病率 prevalence
	流行 epi*
	死亡率 mortality
2. 老年痴呆 (Chinese expressions for dementia 1) AND	发病率 incidence
	发生率 incidence
	患病率 prevalence
	流行 epi*
	死亡率 mortality
3. 老年期痴呆 (Chinese expressions for dementia 2) AND	发病率 incidence
	发生率 incidence
	患病率 prevalence
	流行 epi*
	死亡率 mortality
4. 老年性痴呆 (Chinese expressions for dementia 3) AND	发病率 incidence
	发生率 incidence
	患病率 prevalence
	流行 epi*
	死亡率 mortality
5. 早老性痴呆 (Chinese expressions for dementia 4) AND	发病率 incidence
	发生率 incidence
	患病率 prevalence
	流行 epi*
	死亡率 mortality
6. Alzheimer	发病率 incidence
	发生率 incidence
	患病率 prevalence
	流行 epi*
	死亡率 mortality

Notes: * is used to find singular and plural forms of words and variant endings.

Databases

CNKI

TKA= ('Alzheimer'+ 'dementia'+ 'dementia aged'+ 'presenile dementia') AND TKA = ('incidence'+' prevalence '+' Epidemiology '+' mortality ')

Wanfang

theme:(" Alzheimer " OR " dementia " OR " dementia aged " OR " presenile dementia ") and theme:(" incidence " OR " prevalence " " Epidemiology " OR " mortality ")

PubMed

#1"Dementia"[Mesh]

#2"Alzheimer disease"[Mesh]

#3Dementi*[Title/Abstract] OR Alzheimer*[Title/Abstract] OR Amentia*[Title/Abstract] OR Alzeimer*[Title/Abstract] OR diffuse cortical sclerosis[Title/Abstract]

#4 #1 OR #2 OR #3

#5"incidence"[Mesh]

#6"mortality"[Mesh]

#7"prevalence"[Mesh]

#8"Epidemiology"[Mesh]

#9"Epidemics"[Mesh]

#10incidence*[Title/Abstract] OR Attack Rate[Title/Abstract] OR Person-time Rate[Title/Abstract] OR Attack Rates[Title/Abstract] OR mortalit*[Title/Abstract] OR Fatality[Title/Abstract] OR Death[Title/Abstract] OR prevalence*[Title/Abstract] OR trend[Title/Abstract] OR disease outbreaks[Title/Abstract] OR epidemi*[Title/Abstract]

#11#5 OR #6 OR #7 OR #8 OR #9 OR #10

#12#4 AND #11

#13"China"[Mesh]

#14China[Title/Abstract]

#15#13 OR #14

#17 #15 AND #12

Limit time: 2010-2020

Embase

#1 'Alzheimer disease'/exp

#2 'Dementia'/exp

#3'Dementi*':ti,ab OR 'Amentia*':ti,ab OR 'Alzheimer*':ti,ab OR 'Alzeimer*':ti,ab OR

'diffuse cortical sclerosis':ti,ab

#4 #1 OR #2 OR #3

#5'incidence'/exp

#6'mortality'/exp

#7'prevalence'/exp

#8'attack rate'/exp

#9'epidemic'/exp

#10'Epidemiology'/exp

#11'incidence*':ti,ab OR 'Attack Rate':ti,ab OR 'Person-time Rate':ti,ab OR 'Attack Rates':ti,ab OR 'mortalit*':ti,ab OR 'Fatality':ti,ab OR 'Death':ti,ab OR 'prevalence*':ti,ab OR 'trend':ti,ab OR 'disease outbreaks':ti,ab OR 'epidemi*':ti,ab

#12 #5 OR #6 OR #7 OR #8 OR #9 OR #10 OR #11

#13 #4 AND #12

#14'China'/exp

#15'China':ti,ab

#16#14 OR #15

#17 #16 AND #13

Limit time: 2010-2020

SCOPUS

(TITLE-ABS-KEY (Dementi*) OR TITLE-ABS-KEY (Amentia*) OR TITLE-ABS-KEY (Alzheimer*) OR TITLE-ABS-KEY (Alzeimer*) OR TITLE-ABS-KEY (alzheimer*) OR TITLE-ABS-KEY (diffuse cortical sclerosis)) AND (TITLE-ABS-KEY (incidence*) OR TITLE-ABS-KEY (Attack Rate) OR TITLE-ABS-KEY (Person-time Rate) OR TITLE-ABS-KEY (Attack Rates) OR TITLE-ABS-KEY (mortalit*) OR TITLE-ABS-KEY (Fatality) OR TITLE-ABS-KEY (Death) OR TITLE-ABS-KEY (prevalence*) OR TITLE-ABS-KEY (trend) OR TITLE-ABS-KEY (disease outbreaks) OR TITLE-ABS-KEY (epidemi*)) AND TITLE-ABS-KEY (china) PUBYEAR > 2009 AND PUBYEAR <2021

File SII. Full list of included studies

NO.	References of included studies
**1.**	Bo, Z., Wan, Y., Meng, S. S., Lin, T., Kuang, W., Jiang, L., & Qiu, P. (2019). The temporal trend and distribution characteristics in mortality of Alzheimer's disease and other forms of dementia in China: Based on the National Mortality Surveillance System (NMS) from 2009 to 2015. *PloS one*, *14*(1), e0210621.
**2.**	Cheng, Q., Sun, H. X., Ye, F. L., Wang, G., Ling, H. W., Chen, S. D., & Jiang, G. X. (2014). Dementia among elderly in Shanghai suburb: a rural community survey. *Journal of Alzheimer's Disease*, *39*(4), 883-889.
**3.**	Deng, J., Cao, C., Jiang, Y., Peng, B., Wang, T., Yan, K., ... & Wang, Z. (2018). Prevalence and effect factors of dementia among the community elderly in Chongqing, China. *Psychogeriatrics*, *18*(5), 412-420.
**4.**	Ding, D., Zhao, Q., Wu, W., Xiao, Z., Liang, X., Luo, J., & Hong, Z. (2020). Prevalence and incidence of dementia in an older Chinese population over two decades: The role of education. *Alzheimer's & Dementia*, *16*(12), 1650-1662.
**5.**	Ge, X., Wang, M., Tang, J., Zhou, W., & Xie, Z. (2014). Investigation on the prevalence and risk factors of senile dementia in Changsha area. *Chinese Journal of Nervous and Mental Diseases, 40*(8), 493-496.<br>葛小平，王民主，唐江萍，邹文华, & 谢泽夫. (2014). 长沙地区老年期痴呆患病率及危险因素调查. *中国神经精神疾病杂志*, *40*(8), 493-496.
**6.**	Guan, J. (2018). Incidence of Alzheimer in Persons over 60 Years Old in Taizhou City and Influencing Factors. *Medicine and Society, 31*(6), 52-54.<br>管君花. (2018). 台州市 60 岁以上人群老年痴呆发病情况及影响因素. *医学与社会*, *31*(6), 52-54.
**7.**	Hu, W., Zhou, X., Gong, Z., Zhang, T., Qin, W., & Luo, X. (2018). Mortality analysis of senile dementia in Kunshan from 2005 to 2015. *Chinese Journal of Health Statistics, 35*(4), 542-545.<br>胡文斌，周晓明，巩宗林，张婷，秦威, & 罗晓明. (2018). 2005-2015 年昆山市老年痴呆死亡率分析. *中国卫生统计*, *35*(4), 542-545.
**8.**	Huang Fu. Y., Chang, F., Zhang, F., Hao, X., Guo, J., & Han, L. (2019). Study on the prevalence of senile dementia and its influencing factors in Zhengzhou. *Modern Preventive Medicine, 46*(18), 3347-3350.<br>皇甫赟，常方方，张凤娟，郝习，郭建新, & 韩雷. (2019). 郑州市老年期痴呆患病率及危险因素分析. *现代预防医学*, *46*(18), 3347-3350.
**9.**	Huang, F., Shang, Y., Luo, Y., Wu, P., Huang, X., Tan, X., ... & Hu, X. (2016). Lower prevalence of Alzheimer's disease among Tibetans: association with religious and genetic factors. *Journal of Alzheimer's Disease*, *50*(3), 659-667.
**10.**	Ji, Y., Shi, Z., Zhang, Y., Liu, S., Liu, S., Yue, W., ... & Wisniewski, T. (2015). Prevalence of dementia and main subtypes in rural northern China. *Dementia and geriatric cognitive disorders*, *39*(5-6), 294-302.
**11.**	Jia, L., Du, Y., Chu, L., Zhang, Z., Li, F., Lyu, D., ... & Qiu, Q. (2020). Prevalence, risk factors, and management of dementia and mild cognitive impairment in adults aged 60 years or older in China: a cross-sectional study. *The Lancet Public*

	Health, *5*(12), e661-e671.
**12.**	Kang, M., Gao, Y., Huo, H., Chen, Y., Wang, J., Li, M., & Du, T. (2011). Epidemiological leatures of chronic and Aizheimer'S diseases in the community-based elderly living in cities and couties in Hebei province. *Chinese Journal of Epidemiology, 32*(7), 672-675.<br>康美玉, 高玉梅, 霍红旗, 陈育民, 王健, 李梅杰, & 杜涛. (2011). 河北省 3632 名城乡社区老年人慢性病及老年痴呆的现况调查. *中华流行病学杂志*, *32*(7), 672-675.
**13.**	Lao, M., Zhang, H., Yi, X., Huang, Y., Wu, Z., & Luo, G. (2011). Epidemiological investigation of Alzheimer's disease in Hainan Island. *Chinese Journal of Gerontology, 31*(020), 4016-4018.<br>劳梅丽, 张海英, 易西南, 黄奕弟, 吴志虹, & 罗刚等. (2011). 海南岛阿尔茨海默病的流行病学调查. *中国老年学杂志, 31*(020), 4016-4018.
**14.**	Liao, J., Hunag, H., Yan, J., Ma, J., Tao, X., Liao, X., & Wu, L. (2015). The prevalence of Alzheimer's disease in Nanchang community and its influencing factors. *Chinese Journal of Gerontology,* (24), 7176-7177.<br>廖君, 黄河浪, 闫冀, 马俊, 陶雪琴, 廖雄, & 吴磊. (2015). 南昌市社区老年性痴呆患病率及其影响因素. *中国老年学杂志*, (24), 7176-7177.
**15.**	Ma, Y., Jiang, Z., Wang, J., Xue, J., He, M., & Zhang, Y. (2013). Prevalence of major subtypes of dementia in people over 65 years old in Xujiahui Street, Shanghai. *Chinese Journal of Gerontology, 33*(6), 1365-1366.<br>马勇, 蒋中平, 王继伟, 薛晶晶, 贺明, & 张源等. (2013). 上海徐家汇街道 65 岁以上人群痴呆主要亚型的患病率，*中国老年学杂志，33*(6), 1365-1366.
**16.**	Wu, Y., Cheng, Z., Bao, Z., Fan, J., Tang, L., & Guo, T. (2017). Survey on the status of Alzheimer's disease and psychological health of family caregivers in Wuxi. *Chinese Preventive Medicine, 18*(11), 7.<br>吴越, 程灶火, 包炤华, 范洁, 汤莉, & 过婷等. (2017). 无锡市阿尔茨海默病患病现状和家属照料者心理状况调查研究. *中国预防医学杂志, 18*(11), 7.
**17.**	Yang, L., Jin, X., Yan, J., Jin, Y., Yu, W., Wu, H., & Xu, S. (2016). Prevalence of dementia, cognitive status and associated risk factors among elderly of Zhejiang province, China in 2014. *Age and ageing*, *45*(5), 708-712.
**18.**	Yang, C., Chen, J., Wu, M., Pan, Y., Wang, N., Wu, L., & Huang, G. (2016). Investigation and analysis of the incidence of Alzheimer′s disease in Nanchang community. *Chinese Journal of Gerontology, 36*(22), 5709-5711.<br>杨晨辉, 陈静华, 吴明洋, 潘友根, 王乃博, 吴磊, & 黄国梅. (2016). 南昌市部分社区老年人阿尔茨海默病发病率. *中国老年学杂志*, *36*(22), 5709-5711.
**19.**	Yu, Y., Zhang, N., Dong, J., Lou, Y., & Xiao, W. (2012). Prevalence of Dementia among the Elderly and Its Influential Factors. *Chinese General Practice, 15*(23), 2685-2687.<br>于焰, 张娜, 董佳梅, 娄毅, & 肖卫忠. (2012). 社区老老年人群痴呆患病率及其影响因素研究. *中国全科医学*, *15*(23), 2685-2687.

File SIII. PRISMA Checklist

Section and Topic	Item #	Checklist item	Location where item is reported
**TITLE**			
Title	1	Identify the report as a systematic review.	1
**ABSTRACT**			
Abstract	2	See the PRISMA 2020 for Abstracts checklist.	1
**INTRODUCTION**			
Rationale	3	Describe the rationale for the review in the context of existing knowledge.	1-4
Objectives	4	Provide an explicit statement of the objective(s) or question(s) the review addresses.	4
**METHODS**			
Eligibility criteria	5	Specify the inclusion and exclusion criteria for the review and how studies were grouped for the syntheses.	4
Information sources	6	Specify all databases, registers, websites, organisations, reference lists and other sources searched or consulted to identify studies. Specify the date when each source was last searched or consulted.	4-5
Search strategy	7	Present the full search strategies for all databases, registers and websites, including any filters and limits used.	4-5
Selection process	8	Specify the methods used to decide whether a study met the inclusion criteria of the review, including how many reviewers screened each record and each report retrieved, whether they worked independently, and if applicable, details of automation tools used in the process.	5
Data collection process	9	Specify the methods used to collect data from reports, including how many reviewers collected data from each report, whether they worked independently, any processes for obtaining or confirming data from study investigators, and if applicable, details of automation tools used in the process.	5
Data items	10a	List and define all outcomes for which data were sought. Specify whether all results that were compatible with each outcome domain in each study were sought (e.g. for all measures, time points, analyses), and if not, the methods used to decide which results to collect.	5
	10b	List and define all other variables for which data were sought (e.g. participant and intervention characteristics, funding sources). Describe any assumptions made about any missing or unclear information.	5

Section and Topic	Item #	Checklist item	Location where item is reported
Study risk of bias assessment	11	Specify the methods used to assess risk of bias in the included studies, including details of the tool(s) used, how many reviewers assessed each study and whether they worked independently, and if applicable, details of automation tools used in the process.	5
Effect measures	12	Specify for each outcome the effect measure(s) (e.g. risk ratio, mean difference) used in the synthesis or presentation of results.	5-6
Synthesis methods	13a	Describe the processes used to decide which studies were eligible for each synthesis (e.g. tabulating the study intervention characteristics and comparing against the planned groups for each synthesis (item #5)).	6
	13b	Describe any methods required to prepare the data for presentation or synthesis, such as handling of missing summary statistics, or data conversions.	6
	13c	Describe any methods used to tabulate or visually display results of individual studies and syntheses.	6
	13d	Describe any methods used to synthesize results and provide a rationale for the choice(s). If meta-analysis was performed, describe the model(s), method(s) to identify the presence and extent of statistical heterogeneity, and software package(s) used.	6
	13e	Describe any methods used to explore possible causes of heterogeneity among study results (e.g. subgroup analysis, meta-regression).	6
	13f	Describe any sensitivity analyses conducted to assess robustness of the synthesized results.	N/A
Reporting bias assessment	14	Describe any methods used to assess risk of bias due to missing results in a synthesis (arising from reporting biases).	5
Certainty assessment	15	Describe any methods used to assess certainty (or confidence) in the body of evidence for an outcome.	N/A
**RESULTS**			
Study selection	16a	Describe the results of the search and selection process, from the number of records identified in the search to the number of studies included in the review, ideally using a flow diagram.	6
	16b	Cite studies that might appear to meet the inclusion criteria, but which were excluded, and explain why they were excluded.	6
Study characteristics	17	Cite each included study and present its characteristics.	6-7

Section and Topic	Item #	Checklist item	Location where item is reported
Risk of bias in studies	18	Present assessments of risk of bias for each included study.	7
Results of individual studies	19	For all outcomes, present, for each study: (a) summary statistics for each group (where appropriate) and (b) an effect estimate and its precision (e.g. confidence/credible interval), ideally using structured tables or plots.	8-9
Results of syntheses	20a	For each synthesis, briefly summarise the characteristics and risk of bias among contributing studies.	8-9
	20b	Present results of all statistical syntheses conducted. If meta-analysis was done, present for each the summary estimate and its precision (e.g. confidence/credible interval) and measures of statistical heterogeneity. If comparing groups, describe the direction of the effect.	8-9
	20c	Present results of all investigations of possible causes of heterogeneity among study results.	8
	20d	Present results of all sensitivity analyses conducted to assess the robustness of the synthesized results.	N/A
Reporting biases	21	Present assessments of risk of bias due to missing results (arising from reporting biases) for each synthesis assessed.	7
Certainty of evidence	22	Present assessments of certainty (or confidence) in the body of evidence for each outcome assessed.	N/A
**DISCUSSION**			
Discussion	23a	Provide a general interpretation of the results in the context of other evidence.	9-12
	23b	Discuss any limitations of the evidence included in the review.	11
	23c	Discuss any limitations of the review processes used.	11
	23d	Discuss implications of the results for practice, policy, and future research.	11-12
**OTHER INFORMATION**			
Registration and protocol	24a	Provide registration information for the review, including register name and registration number, or state that the review was not registered.	4
	24b	Indicate where the review protocol can be accessed, or state that a protocol was not prepared.	4
	24c	Describe and explain any amendments to information provided at registration or in the protocol.	4
Support	25	Describe sources of financial or non-financial support for the review, and the role of the funders or sponsors in the review.	N/A

Section and Topic	Item #	Checklist item	Location where item is reported
Competing interests	26	Declare any competing interests of review authors.	Title page
Availability of data, code and other materials	27	Report which of the following are publicly available and where they can be found: template data collection forms; data extracted from included studies; data used for all analyses; analytic code; any other materials used in the review.	2
